# Positive and Negative Effects of Metal Oxide Nanoparticles on Antibiotic Resistance Genes Transfer

**DOI:** 10.3390/antibiotics9110742

**Published:** 2020-10-27

**Authors:** Georgy D. Otinov, Alina V. Lokteva, Anastasia D. Petrova, Irina V. Zinchenko, Maria V. Isaeva, Evgeny A. Kovtunov, Elena I. Koshel

**Affiliations:** Microbiology Lab of SCAMT Institute, ITMO University, 197101 St. Petersburg, Russia; otinov@scamt-itmo.ru (G.D.O.); lokteva@scamt-itmo.ru (A.V.L.); petrova@scamt-itmo.ru (A.D.P.); zinchenko@scamt-itmo.ru (I.V.Z.); isaeva@scamt-itmo.ru (M.V.I.); kovtunovea@mail.ru (E.A.K.)

**Keywords:** metal oxide nanoparticles, horizontal gene transfer, antibiotic resistance, conjugation, transformation

## Abstract

Rapid development of antibiotic resistance in bacteria is a critical public health problem in the world. One of the main routes of resistance development is the transfer of genes containing antibiotic resistance cassettes. Gene transfer can be done through horizontal transfer of genes: transduction, conjugation, and transformation. Many factors in the environment influence these processes, and one of them is the action of metal oxide nanoparticles (MONPs), which can appear in the milieu through both biological synthesis and the release of engineered nanomaterial. In this study, the effect of AlOOH, CuO, Fe_3_O_4_, TiO_2_, and ZnO MONPs on the transformation (heat shock transformation) of bacteria *Escherichia coli* K12, and the conjugation between *E. coli* cc118 and *E. coli* Nova Blue were studied. The MONPs were synthesized by one method and fully characterized. ZnO nanoparticles (NPs) have significantly increased the efficiency of transformation (more than 9-fold), while the other NPs have reduced it to 31 times (TiO_2_ NPs). AlOOH NPs increased the number of transconjugants more than 1.5-fold, while CuO and Fe_3_O_4_ NPs did not have a significant effect on transformation and conjugation. Thus, the data shows that different types of MONPs can enhance or inhibit different gene transfer mechanisms, affecting the spread of antibiotic resistance genes.

## 1. Introduction

The development of microbial resistance to antimicrobial agents is a complex and urgent problem of biomedicine. The emergence and spread of antibiotic-resistant bacterial strains are among the most acute public health problems globally, as identified by the World Health Organization [1]. This rapid development of the resistance is associated with the increased use of antibiotics in medicine, agriculture, and animal feedstuffs [2].

The primary way of antibiotic resistance development is the transport of antibiotic resistance genes (ARGs) in the environment (such as soil [3], water [4], and air [5]), which can be provided by mobile genetics elements, such as plasmids [6]. ARGs can be transferred by lateral gene transfer (or horizontal gene transfer (HGT)) [7]. There are several HGT processes, namely conjugation, transduction, and transformation [8,9]. In the process of conjugation, DNA is transferred from cell to cell; during the transduction, DNA is provided by bacteriophage; in the process of the transformation, extracellular DNA is introduced from the environment, for which the cells should be competent [10].

The significant factors that influence HGT are environmental, which, for example, can affect the natural competence of bacteria [10]. One of them is metal oxides nanoparticles (MONPs), which are one of the most reactive phases in the natural environment and could originate extensively through either natural processes or the release of engineered nanomaterials [11].

A few papers have shown the effect of MONPs on increasing or decreasing HGT [12,13,14,15,16,17,18,19]. Thus, mainly the effect of MONPs on gene transfer is associated with the formation of reactive oxygen species (ROS) [13,14,15], as well as with the direct interaction of MONPs with the DNA matrix, which plays an important role when the matrix is in the environment [11]. MONPs can also affect central metabolism, the respiration process [16,17,18], and ROS active production can lead to DNA damage, which causes the SOS response, which may also promote HGT [19]. Existing data is contradictable and does not clearly understand the MONPs action mechanisms due to the significant differences between particles parameters. These differences occur mainly because of variations in synthesis [11,13,20,21,22]. Our study used MONPs, which were synthesized under the identical condition by the sol-gel method and fully characterized to avoid variation issues. This allows for a relevant comparison of the effects of MONPs on HGT.

In this study, we consider the following nanoparticles (NPs): boehmite (AlOOH), copper oxide (CuO), magnetite (Fe_3_O_4_), titanium dioxide (TiO_2_), and zinc oxide (ZnO) as representative MONPs since they are the most widely produced and used NPs worldwide. For example, ZnO NPs and CuO NPs are widely used in medicine as antibacterial agents [23,24,25]; Fe_3_O_4_ NPs for targeting drug delivery and diagnostic application [26,27]; and aluminum compounds are used in vaccine development, for example, aluminum adjuvants [28]. TiO_2_ NPs has found wide application in cosmetology [29].

Thus, objective of this study was to examine whether MONPs influence horizontal ARGs transfer. For transformation by a pGEM-T plasmid, we chose *Escherichia coli* K12 wild type, which does not have mutations that increase the efficiency of transformation (for example, in the *rec^−^* and *endA^−^* genes [10,30]) as the model organism. Moreover, we used heat-shock transformation as the laboratory model of the natural transformation process, as it was previously applied by other researchers [11,13,22]. The conjugation was investigated by the transfer of plasmid pKNG101 from *E. coli* cc118 to *E. coli* Nova Blue. The MONPs were added to the *E. coli* cultivation medium for transformation and conjugation. As a result, the different types of MONPs can enhance or inhibit different gene transfer mechanisms, affecting the spread of antibiotic resistance genes.

## 2. Results

### 2.1. MONPs Antibacterial Activity

Some MONPs have a significant antibacterial activity that can be compared to antibiotics [31,32,33,34], and this ability may influence HGT.

**Figure 1:**
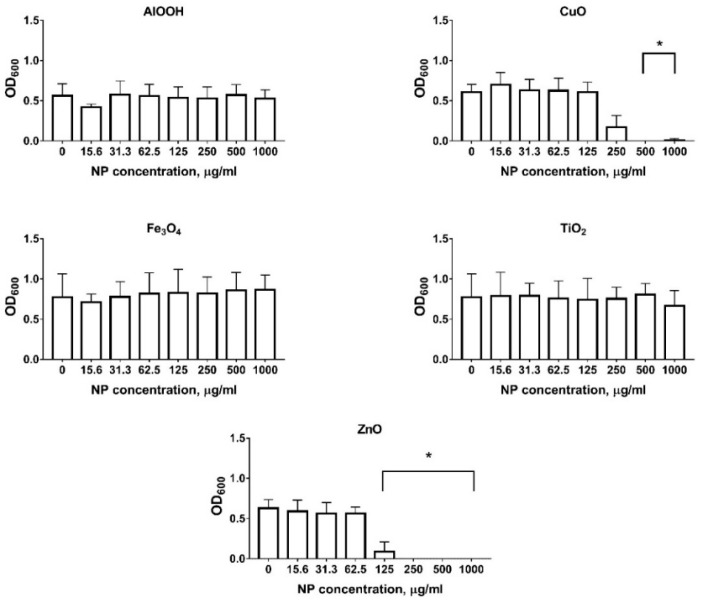
Metal oxide nanoparticles (MONPs) antibacterial activity against *E. coli* K12. The presented results are the mean of three independent experiments (three replicates in each) ± standard deviation. * *p* < 0.05.

As shown in Figure 1, the majority of MONPs, namely AlOOH, Fe_3_O_4_, and TiO_2_, do not inhibit *E. coli* K12 growth. However, CuO and ZnO NPs have a significant antibacterial effect when their concentration is above 100 µg/mL for ZnO and 125 µg/mL for CuO. ZnO inhibits growth at 250 µg/mL. At the same time, the value for CuO NPs are 500 µg/mL. Thus, to further investigate the effect of NPs on HGT, we consider concentrations for ZnO and CuO NPs less than their inhibitory concentrations.

### 2.2. Effect of MONPs on Transformation

MONPs have been added to the cultures in concentrations 50 µg/mL for the transformation of *E. coli* K12 by pGEM-T plasmid containing cassette resistance to ampicillin (Amp^R^).

**Figure 2:**
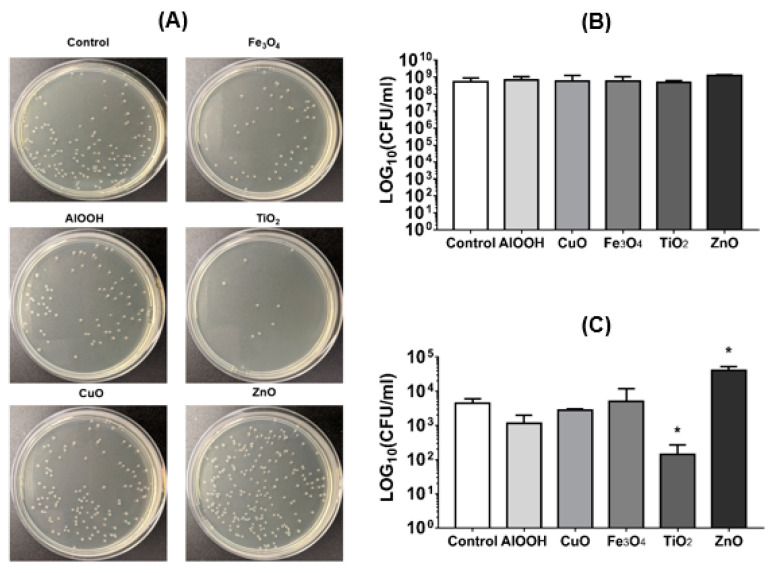
Effect of MONPs on transformation. (**A**) Images of bacteria colony-forming units (CFU) of transformants on lysogeny broth (LB)-agar plates with ampicillin (100 µg/mL) after transformation by pGEM-T plasmid in MONPs presence (50 µg/mL). (**B**,**C**) Statistical analysis of the CFU number in the presence of MONPs (50 µg/mL) before transformation (**B**) and after transformation (**C**). The presented results are the mean of three independent experiments (three replicates in each) ± standard deviation. * *p* < 0.05.

The transformation resulted in the production of bacteria resistant to ampicillin through the transfer of a pGEM-T plasmid containing an ampicillin-resistant cassette (Figure 2A). Even though TiO_2_ NPs do not influence on bacterial growth (Figure 2B), they significantly reduced the efficiency of transformation by more than 31 times (Figure 2A,C). At the same time, in the presence of ZnO, the colony-forming units (CFU) number of transformants was more than 9-fold higher compared to the control group without NPs (Figure 2A,C). AlOOH, CuO, and Fe_3_O_4_ NPs do not have a significant effect on the transformation (Figure 2C and Table 1).

### 2.3. Effect of MONPs on Conjugation

To study conjugation, *E. coli* cc118 with a plasmid containing a cassette resistance to streptomycin Sm^R^ was chosen as a donor and *E. coli* Nova Blue with a cassette resistance to tetracycline Tet^R^ as a recipient. MONPs were used in two concentrations –50 µg/mL for AlOOH, CuO, Fe_3_O_4_, TiO_2_, and ZnO, 500 µg/mL for AlOOH, Fe_3_O_4_, and TiO_2_, because CuO and ZnO are toxic for cells at concentrations 500 µg/mL, as shown above.

**Figure 3:**
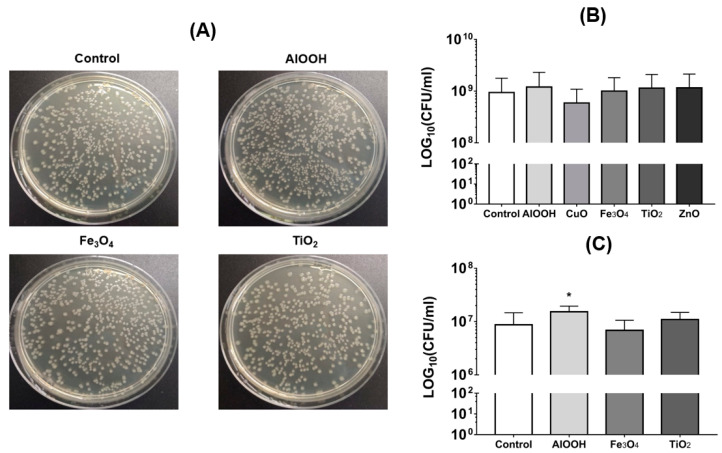
Effect of MONPs on conjugation. (**A**) Images of bacteria CFU of transconjugants on LB-agar plates with streptomycin (100 µg/mL) and tetracycline (12.5 µg/mL) after conjugation in MONPs presence (500 µg/mL). (**B**,**C**) Statistical analysis of the CFU number after conjugation in the presence of MONPs in concentration (**B**) 50 µg/mL, (**C**) 500 µg/mL. The presented results are the mean of three independent experiments (three replicates in each) ± standard deviation. * *p* < 0.05.

*E. coli* cultures resistant to two antibiotics: tetracycline and streptomycin, were obtained in this experiment (Figure 3A). As presented in Figure 3B and Table 1, MONPs in concentrations of 50 µg/mL do not significantly affect conjugation. In concentrations of 500 µg/mL (Figure 3A,C), AlOOH had a significant effect on the conjugation—increasing the number of transconjugants by 1.8 times. In the case of other MONPs, they do not have a statistically significant effect on the conjugation process (Figure 3C and Table 1).

## 3. Discussion

In this study, we have shown that MONPs can have different effects on antibiotic resistance gene transfer. We studied the effect of MONPs on a wide-spreading line of MONPs, which were synthesized using one method, identical conditions, and fully characterized (Table 2). This is important as the synthesis method affects the physical and chemical characteristics of MONPs, which, in turn, determine the biological properties of the particles [13,21,35]. Thus, in this study, we were able to correctly compare the influence of different MONPs on gene transfer.

During the transformation, DNA should pass through the cell wall, which plays a semi-permeable barrier. One of the properties of MONPs is to induce the generation of ROS. These agents can cause disorganizing and damaging of bacterial cell walls and membranes [14,20,36]. Moreover, possibly due to the long-term interaction of NPs with bacteria, their metabolism changes [17,18]. Subsequent processing of bacteria by transformation buffer (TB) leads to even more significant changes in the cell wall and, as a result, increased ability to absorb extracellular DNA. As a result, this leads to the altering cell membrane penetration and increased uptake of ARGs [13].

Our data showed that ZnO NPs enhances the transformation up to 9.1 times. These results are consistent with data by Wang et al. [13] but contradict with results by Hu et al. [11]. The difference may be due to the use of another protocol in our study. We used overnight pre-incubation of bacteria with MONPs before the transformation, and that did not affect the cell viability, but, perhaps, appears to have affected bacterial competence. The increase in competence may be related to the release of zinc ions in lysogeny broth (LB). It is shown that the effect of zinc ions on the number of transformants is higher than that of NPs; however, in that study, NPs were contained in LB agar, and they could not release cations sufficiently [16]. In our work, cultivation was conducted in a liquid medium that provided enough zinc ions production. The positive influence of ZnO on transformants CFU may also be explained by the ability to increase cell membrane permeability, leading to increased transformant CFU [13].

TiO_2_ NPs have reduced the efficiency of transformation up to 31 times. The decrease in the number of transformants is most probably determined by the interaction of NPs with external DNA. This binding is achieved through non-covalent bonds, i.e., the attraction of positively charged particles and a negatively charged phosphate backbone [11]. However, the suppression of transformation by TiO_2_ is much more significant than by AlOOH (Table 1), while zeta potential AlOOH is several times higher than TiO_2_ (Table 2). The possible differences in the number of transformants can also be attributed to the more active production of ROS by TiO_2_ particles. It can also be assumed that TiO_2_ NPs have a substantial effect on changing metabolism [17]. It was shown that long incubation *Bacillus subtilis* with TiO_2_ NPs significantly reduces the number of transformants [16]. The impact of all factors (DNA bindings, ROS products, metabolic changes) is certainly possible, which leads to such a significant reduction in CFU transformants.

In the case of AlOOH, the NPs effect might have an electrostatic interaction with the *E. coli* K12 cell wall [37] due to its high zeta potential (Table 2). AlOOH on the surface of the cell wall can also interact with DNA, leading to the formation of NPs-DNA agglomerates. During heat-shock transformation, these agglomerates cannot enter the cell due to their large size [11]. This may lead to a reduction of CFU transformation.

Earlier, it was shown by us [31] and colleagues [14,15,20] that aluminum and copper oxide NPs have a positive effect on the number of transconjugants. It can be explained as AlOOH NPs have high zeta potential (Table 2), because conjugation efficiency can be improved by adsorption of NPs on the bacterial membrane surface, reducing the distance between donor and recipient, thereby increasing the conjugation frequency [38].

Thus, the comparison analysis showed differences in the influence of MONPs on different gene transfer mechanisms. MONPs have different effects on transformation and conjugation, most likely due to their physical and chemical characteristics. The exact mechanisms of these effects have to be clarified.

## 4. Materials and Methods

### 4.1. Chemicals

MONPs (AlOOH, CuO, Fe_3_O_4_, TiO_2_, and ZnO) were obtained by sol-gel synthesis. The synthesis process of the MONPs and their characteristics were previously described [21]. Hydrodynamic size, zeta potential, and surface parameters of NPs are presented in Table 2.

### 4.2. Bacterial Strains

*E. coli* K12 wild type, *E. coli* Nova Blue (endA1 hsdR17 (r_K12_^–^ m_K12_^+^) supE44 thi-1 recA1gyrA96 relA1 lac Fʹ[proA+B+lacIqZΔM15::Tn10]) (Tet^R^) (Novagen, Darmstadt, Germany) and *E. coli* cc118 ∆(ara, leu) araD ∆lacX 74 galE galK PhoA20 thi-1 rpsE rpoB argE (am) recA1, Sm^R^ with plasmid pKNG 101 (6986 bp) contains a streptomycin cassette resistance (Sm^R^) [39]. *E. coli* strains were grown at 37 °C in lysogeny broth (LB) medium supplemented with appropriate antibiotics: 100 μg/mL of ampicillin, 12.5 μg/mL of tetracycline, 100 μg/mL of streptomycin (all used antibiotics were provided by PanReac AppliChem, Barcelona, Spain).

### 4.3. MONPs Antibacterial Activity

To select the optimal MONPs concentrations that are non-toxic to *E. coli*, we first studied the antibacterial properties of the particles. For this CFU 10^9^ overnight culture of *E. coli* K12 was diluted ten times in a suitable media with AlOOH, CuO, Fe_3_O_4_, TiO_2_, and ZnO in concentrations from 15.6 µg/mL to 1 mg/mL, and 200 µL of each solution was inoculated into 96-well plates. Samples were incubated for 24 h. Optical density at a wavelength of 600 nm (OD_600_) was measured using a microplate reader Infinite F50 (TECAN, Mennedorf, Switzerland)); the bactericidal properties of the particles were evaluated. The positive control was a strain in a nutrient medium without the addition of particles. Negative controls were the medium with appropriate concentrations of MONPs without the addition of bacterial cells.

### 4.4. Transformation Test

*E. coli* K12 was cultivated in LB broth under 250 rpm at 37 °C overnight. After the bacteria cells were diluted in ratio 1:1000 into fresh LB broths with AlOOH, CuO, Fe_3_O_4_, TiO_2_, and ZnO NPs in concentrations 50 µg/mL and cultivated under the conditions described below for 16–18 h.

A total of 1 mL of each culture was transferred to a sterile ice-cold 1.5 mL polypropylene tube, centrifuged at 8000 rpm for 5 min at 4 °C, and re-suspended in 1 mL ice-cold transformation buffer (2.4 g/L HEPES; 10.8 g/L MnCl_2_∙4H_2_O; 1.74 g/L CaCl_2_; and 18.6 g/L KCl). Then cells were kept on an ice-bath for 10 min, recovered by centrifugation under subsequently described conditions, re-suspended in 50 µL TB buffer. All of the cells volume was mixed with 213 ng of the pGEM-T plasmid containing cassette resistance to ampicillin (Amp^R^) (Promega, Madison, Wisconsin, USA), the size of which is 3000 bp, and the plasmid contains the cassette resistance to ampicillin. The suspension incubated on an ice-bath for 15–20 min, then the tubes were transferred to a rack placed in preheated 42 °C Biosan CH-100 (Biosan, Riga, Latvia) and stored for 30 s. After tubes were returned to the ice bath and left to chill for 2 min, 250 µL of super optimal broth with catabolite repression (SOC) medium was added to the tubes. The cells were incubated for one hour at 37 °C, transformed competent cells were seeded on the LB agar containing 100 µg/mL ampicillin to determine CFU.

### 4.5. Conjugation Test

*E. coli* cc118 with conjugative plasmid pKNG101 was chosen as a donor. The plasmid contains a cassette resistance to streptomycin (Sm^R^). The recipient is *E. coli* Nova Blue with the tetracycline resistance (Tet^R^). The strains were incubated in LB broth containing specific antibiotics and shaken overnight under conditions described below. The donor and recipient were then diluted in ratio 1:1000 into fresh LB broth and cultivated at the same conditions for 16–18 h. The cells were washed twice by physiological solution and re-suspended. The conjugation mating system contained the same amount of donor and recipient cells (mixed at 1:1 ratio). The mixes were added to different concentrations of AlOOH, CuO, Fe_3_O_4_, TiO_2_, and ZnO (50 µg/mL and 500 µg/mL each) and incubated with shaking (250 rpm) at 37 °C for 2 h. After that, the tubes were incubated without shaking for 24 h. The number of conjugants was considered by seeding on the LB agar with streptomycin (100 µg/mL) and tetracycline (12.5 µg/mL).

### 4.6. Statistics

Each experiment for the study of antibacterial activity, transformation, and conjugation was carried out three times. Statistical analyses were performed in GraphPad Prism 7.0 software. Statistical significance was considered at *p* < 0.05 and was defined by Kruskal–Wallis test. Data were expressed as mean ± standard deviation.

## 5. Conclusions

In this study, we have shown differences in the effect of MONPs synthesized by one method and fully characterized on ARGs transfer. ZnO NPs increased transformation up to 9.1 times. In the case of TiO_2_ was observed the 31-fold reduction of the transformants number. Other NPs do not significantly influence the transformation process. AlOOH NPs enhanced the conjugation by 1.8 times at 500 µg/mL concentration. Whereas CuO, Fe_3_O_4_, TiO_2_, and ZnO NPs did not have a statistically significant effect on the conjugation. Differences in effects by different MONPs are most likely due to their chemical and physical properties, which is still to be clarified in future studies.

The described double action of MONPs can have both negative consequences and benefits in different areas. On the one hand, considering their active use in various industries, one should reflect on the possible subsequent spread of ARGs. On the other hand, some MONPs have a negative impact on gene transfer and can potentially reduce the risk of antibiotic-resistant strains. Moreover, the protocols described for the MONPs application may also be useful in laboratory practice for transformation and conjugation.

Thus, the data presented in this paper, on the one hand, demonstrate the potential negative consequences of mass application of certain nanomaterials, which is due to the spread of ARGs. On the other hand, our data suggested new possible applications for MONPs in genetic engineering due to their unique properties.

## Figures and Tables

**Table 1:** The effects of MONPs on antibiotic resistance genes transfer calculated as a ratio between experimental and control colony-forming units (CFU) number.

NPs	Concentration, µg/mL	Effect on Transformation	Effect on Conjugation
AlOOH	50	↓ 3.8 times	↑ 1.3 times
	500	-	↑ 1.8 times **
CuO	50	↓ 1.6 times	↓ 1.6 times
Fe_3_O_4_	50	↑ 1.2 times	No effect *
	500	-	↓ 1.3 times
TiO_2_	50	↓ 31.1 times **	↑ 1.2 times
	500	-	↑ 1.3 times
ZnO	50	↑ 9.1 times **	↑ 1.2 times

* The effect is less than 1.1 time. ** Statistically significant difference compared to control (*p* < 0.05).

**Table 2:** Hydrodynamic size, zeta potential, and surface parameters of MONPs.

MONPs	Hydrosol Parameters		Surface Parameters	
	Hydrodynamic Diameter, nm	Zeta Potential, mV	S_BET_, m^2^/g	Pore Size, nm
AlOOH	90 ± 10	+42.0 ± 0.5	170	3.5
CuO	500 ± 50	+10.8 ± 0.4	42	3.3
Fe_3_O_4_	60 ± 20	+30.0 ± 1.2	120	9
TiO_2_	40 ± 7	+7.2 ± 0.3	167	5
ZnO	500 ± 70	+18.0 ± 0.3	20	3
